# LncRNA BCAR4, targeting to miR-665/STAT3 signaling, maintains cancer stem cells stemness and promotes tumorigenicity in colorectal cancer

**DOI:** 10.1186/s12935-019-0784-3

**Published:** 2019-03-27

**Authors:** Shurui Ouyang, Xin Zhou, Zhengquan Chen, Maijian Wang, Xinbin Zheng, Ming Xie

**Affiliations:** Department of Gastrointestinal, The Affiliated Hospital of Zunyi Medical College, Huichuan Area, Dalian Road 149, Zunyi, 563000 Guizhou Province China

**Keywords:** Breast cancer anti-estrogen resistance 4 (BCAR4), Colorectal cancer (CRC), LncRNA, ALDH+ cancer stem/initiating cells, miR-665

## Abstract

**Background:**

Breast cancer anti-estrogen resistance 4 (BCAR4) is closely associated with colorectal cancer (CRC) initiation and propagation. However, the mechanisms underlying BCAR4 function in colon cancer remains largely unknown. In this study, we hypothesized that BCAR4 could regulate colon cancer stem/initiating cells (CSC) function and further facilitates the colon cancer progression.

**Methods:**

qRT-PCR was used to examine the expression of BCAR4 and various CSC markers. FACS, acetaldehyde dehydrogenase (ALDH) activity and western blot assays were applicable to test the expression of CSC markers. CCK8, tumorsphere formation and transwell assays were adopted to examine the capacity of CRC cells proliferation, self-renewal and migration. Pull down assay was used to test the interaction between BCAR4 and miR-665. Luciferase reporter assay was used to examine the interaction of miR-665 and activators of transcription (STAT3). In vivo tumor xenograft study was used to verify the malignancy of CRC cells with inhibition of BCAR4.

**Results:**

Breast cancer anti-estrogen resistance 4 was highly expressed in both CRC cells and stem/initiating cells. In addition, overexpression of BCAR4 facilitated the maintenance of ALDH positive cells (a type of cancer stem/initiating cells) stemness and promoted ALDH+ cells proliferation and migration. Inhibition of BCAR4 restricted ALDH+ cells proliferation and migration. We further proved that miR-665 was the target of BCAR4 and subsequently activated signal transducers and STAT3 signaling which is an important pathway in cancer stem cells self-renewal.

**Conclusions:**

Breast cancer anti-estrogen resistance 4 promotes the CRC cells stemness through targeting to miR-665/STAT3 signaling and identification of the BCAR4 in CRC stem cells provides a new insight into CRC diagnosis, treatment, prognosis and next-step translational investigations.

## Background

Colorectal cancer (CRC), is commonly originated from normal colon epithelial cell. Unwholesome lifestyle and little hereditary mutations are the major risk factors of CRC [1, 2]. More than 1 million people worldwide suffered from threaten of CRC every year [3]. Cytoreductive surgery plus chemotherapy or radiotherapy, as the traditional therapeutic regimens, is widely applicable in CRC treatment [4]. In addition, immunotherapy, as a precision treatment, provided novel insights in CRC cure [5]. Non-specific immunoregulators, such as Bacillus Calmette–Guérin (BCG) and levamisole were initially used in CRC treatments [6]. Subsequently, passive (antibodies or immune cells) and active (vaccination) specific immunotherapy was applicable for CRC and showed limited efficiency [7]. Unfortunately, the mortality of CRC is still increased. Due to high rate of recurrence and high heterogeneity of colorectal cancer, many investigators focus on the cancer stem cells (CSCs) which possess the capacity of self-renewal and differentiation [8]. There were many markers of CSC depended on the cancer types. For example, in colon cancer, cell surface markers such as CD133, CD44, ALDH and ATP-binding cassette sub-family B member 5 (ABCB5) were reported to be a identification of CSCs (21). Unlike the bulk of adult stem cells, CSCs are considered to initiate tumor growth and cause the recurrence of cancer after chemotherapy and/or radiation therapy [9].

Besides, non-coding RNAs, for example long non-coding RNAs (lncRNAs) have been demonstrated to be closely associated with CRC and drawn the most attention until now [10]. LncRNA, contains more than 200 nucleotides, is defined as a transcript which can not translated into proteins [11]. Generally, lncRNA could regulate the cells function as a signaling mediator, decoy molecule, guide and scaffold protein. Especially, lncRNA could interact with miRNA, which is a class of small non-coding RNA and closely associated with various cancer types [12], and further block its downstream signaling pathways. Owing to their versatile capacity and disease-restricted expression, lncRNAs have been proposed to be an attractive biomarker and therapeutic target in cancers [13]. For example, Wang and colleagues characterized the epigenetic landscape of lncRNAs across 6457 tumors and 455 cancer cell lines. They found epigenetically-induced lncRNA1 (EPIC1) was associated with luminal B breast cancer prognosis and promoted tumor growth through regulating MYC which is a proto-oncogene [14]. In addition, Hosono et al. [15] identified a novel lncRNA, THOR, and proved that THOR functioned as an oncogene and promoted tumor progression via interacting with insulin-like growth factor 2 mRNA-binding protein 1 (IGF2BP1). More advanced studies revealed that long non-coding RNA breast cancer anti-estrogen resistance 4 (BCAR4) facilitated colon cancer progression through activating wnt/β-catenin pathway. However, whether and how BCAR4 is implicated in colorectal CSCs remains largely unknown [16, 17].

In this study, we found that BCAR4 was highly expressed in the colorectal cancer tissues. Also, several colon cell lines and especially ALDH+ CSCs showed higher expression of BCAR4. Consequently, colorectal cancer cells (HCT116 and HCT8) with BCAR4 overexpression or inhibition were established to examine its function. Results showed BCAR4 maintained the stemness of CSCs and promoted cancer cells proliferation and migration. Further, miR-665 was demonstrated to be the target of BCAR4 and subsequently activated STAT3 signaling which is an important pathway in cancer stem cells self-renewal. Overall, identification of the BCAR4 in CSCs could be a novel diagnostic marker for CRC as well as provided a new insight into CRC treatment, prognosis and next-step translational investigations.

## Materials and methods

### Patients and tumor samples

Tumor tissues and its adjacent normal tissues derived from 30 colorectal cancer patients at advanced stage were collected for BCAR4 examination. These samples were collected and stored at − 80 °C. We claimed that these patients received no radiotherapy before surgery.

### Cell culture

Human intestinal cancer cells, HCT116,HCT8 and SW480, were purchased from the Bena culture collection Co., Ltd (Jiangsu, China); the normal human colon epithelial cells, CCD 841 CoN, were purchased from Thermo Technology (USA). Cells were cultured according to the manufacturer’s instructions. Briefly, we cultured these cells with Dulbecco’s modified Eagle’s medium (DMEM). Additionally, 10% FBS and 1% penicillin/streptomycin should be added (Gibco, Rockville, MD, USA) for three cell lines culture. Normal colon epithelial cells were also grown in DMEM. And 15% FBS, 4 mM l-glutamine and 1% penicillin/streptomycin is necessary for these cells growth. Cell incubator, with a humidified atmosphere containing 5% CO_2_, is applicable for all cells culture.

### RNA extraction and real-time PCR analysis

The mirVana miRNA kit (Takara, Dalian, China) is applicable to extract total RNA from tumor or normal tissue samples and cultured cells following the manufacturer’s instructions. Of note, internal control that we used is U6 small RNA. For the detection of BCAR4 and other CSCs markers expression, PrimeScript RT reagent kit (Takara, Dalian, China) was applicable for synthesizing the first-strand cDNA. The expression of BCAR4 and CSCs markers were quantified by Real-time PCR Mixture assays (Takara). GAPDH was used as the internal control. The primers of BCAR4 were following: Forward, 5′-A C A G C A G C T T G T T G C T C A T C T-3′, and Reverse, 5′-T T G C C T T G G G G A C A G T T C A C-3′; CD133 primers was forward: 5′-T T C T A T G C T G T G T C C T G G G G C-3′ and Reverse, 5′-T T G T T G G T G C A A G C T C T T C A A G G T-′3. SOX2 primers was Forward: 5′-A G C C C C A A G A T G C A C A A C T C-3′, and Reverse, 5′-C T C C G G G A A G C G T G T A C T T A-3′. ALDH forward 5′-T G G C T G A T T T A A T C G A A A G A G A T-3′, and Reverse, 5′-T C C A C C A T T C A T T G A C T C C A-3′.

### Aldefluor assay

Aldefluor assay was performed according to the manufacturer’s instructions (Aldagen, UK). Briefly, we label one TEST and one CONTROL tube for each sample. Lysed the cells and add activated ALDEFLUOR reagent into lysed cell suspension. Add 5 μl DEAB into control tube and then transferred into TEST tube. Mix and immediately transfer 0.5 ml the mixture to the CONTROL tube. Incubate at 37 °C and centrifuge. Then remove supernatant and resuspend in assay buffer. Finally, flow cytometer was used to acquire data.

### Plasmid construction

The plasmids including pBABE-puro-BCAR4 and pLKO.1-BCAR4 were constructed according to manufacturer’s instructions (sigma, USA). In brief, PCR was applicable to amplify the CDS region of BCAR4 gene and then construct and amplify the pBABE-puro-BCAR4 plasmid (Sigma, USA) in 293T cells. In addition, shBCAR4 was designed to construct pLKO.1-BCAR4 plasmid (Sigma, USA) in 293T cells.

### Cell transfection

Several plasmids, including pBABE-puro-BCAR4 and its corresponding negative control (pBABE-puro-NC), pLKO.1-BCAR4 and its control (pLKO.1-NC) were prepared to transfected into CRC cells. Lipofectamine 2000 (Invitrogen, Carlsbad, CA, USA), a common transfection reagent, was performed to efficiently transfect pBABE-puro-BCAR4 or pLKO.1-BCAR4 in CRC cells according to the manufacturer’s instructions.

### CCK8 assay

We assessed Cell viability through Cell Counting Kit-8 assay (CCK-8) according to the manufacturer’s protocol (Dojindo; Tokyo, Japan). 2 × 10^3^ Cells were seeded in 96-well plates and incubated at 37 °C for 24 h, 48 h or 72 h in a humidified chamber containing 5% CO2. Then, we added the CCK-8 solution (10 μl) into each well, and the plates were incubated for 1 h at 37 °C. The absorbance of cells at 450 nm (OD450) was measured in a microplate reader (Bio-Rad, USA).

### Tumorsphere formation assay

Tumorsphere formation assay was performed following the manufacturer’s instructions (R&D, USA). In brief, after transfection, we seeded 200 cells in tumorsphere medium into a 96-well plate. Next step, seal the upper and lower edges of the 96-well plate. After 1-week incubation, tumorsphere numbers were counted under a phase-contrast microscope.

### ALDH activity assay

ALDH Activity Assay Kit (Abcam, ab155893) was applicable to test ALDH activity of CRC cells with BCAR4 over-expression or inhibition. Briefly, Add NADH standard into a 96 well plate and adjust the volume to 50 μl/well with ALDH Assay Buffer. Then mix the cells and ALDH assay buffer for removing nuclei and insoluble material. Finally, incubate for 20–60 min at room temperature and measure at OD450 nm.

### Transwell migration assay

The capacity of CRC cells migration was determined by Transwell assay (Thermo, USA), In brief, we plated cancer cells on the upper layer of a cell culture insert with permeable membrane. Test agent is placed below the cell permeable membrane. Then we incubated the cells for 3–18 h and counted the cells.

### Pull down assay

Colorectal cancer cells (HCT-116 and HCT-8) were lysed with RIPA lysis buffer (Thermo, USA). Then, we added the biotin-label miR-665 magnetic beads for incubation at 4 °C overnight. The bound RNAs were purified using TRIzol reagent (Invitrogen) for further RT–qPCR analysis.

### Luciferase reporter assay

The human STAT3 3′-UTR consist of miR-665 binding site and a mutant variant were amplified by PCR and then plasmids including STAT3-Wt-3′-UTR and STAT3-Mut-3′-UTR, were constructed. For luciferase assays, HEK293T cells were cultured in a 6-well plate and then co-transfected with the miR-665 or miR-NC (100 nM/well) and STAT3-Wt-3′-UTR reporter plasmid or STAT3-Mut-3′-UTR reporter plasmid (100 ng/well) and the pRL-TK luciferase reporters (25 ng/well) using Lipofectamine 2000 (Invitrogen). Finally, Dual-Luciferase Reporter Assay kit (Promega, Madison, WI, USA) was used to examine the Luciferase activity levels according to the manufacturer’s instructions. Renilla-luciferase was used for normalization.

### Western blot assay

Expression of various CSCs markers were determined by western blot. Briefly, we first lysed the CRC cells with RIPA buffer. Primary antibodies, such as, rat anti CD133 (santa cruze, 1:500) and mouse anti STAT3 (santa cruze, 1:1000) were integrated with the targeted protein with incubation at room temperature for 1–2 h. Second antibodies conjugated with HRP label, were used to detect the expression of STAT3 and CD133 through chemiluminescence reagent. Mouse actin was used as the loading control.

### In vivo tumor xenograft study

Four-week-old male nude mice (Vital River Laboratory Animal Technology, China) with immune deficiency were used under conditions approved by The Affiliated Hospital of Zunyi Medical College. To assess the inhibited effect of LOF BCAR4 on tumor growth, 2 × 10^6^ ALDH+ HCT-8 cells transfected by pLKO.1-BCAR4, were suspended in 0.2 ml sterile saline and subsequently implanted subcutaneously into the axillary fossae of each mouse. After 2 week implantation, we killed all mice and removed xenograft tumors intactly. The volume of xenograft tumors was calculated as follows: length × width2 × 1/2. And fresh tissues from xenograft tumors were immediately snap frozen in liquid nitrogen and stored at − 80 °C.

### Data analysis

Data are performed as mean ± standard deviation. Statistical analyses between two groups were performed using Student’s t-test via SPSS 16.0 (Chicago, IL, USA). However, statistical analyses between multiple groups were performed using one way analysis of variance followed by the least significant difference post hoc test. Differences with values of *P *< 0.05 were regarded as statistically significant. Each experiment was performed independently at least for 3 times.

## Results

### BCAR4 was significantly up-regulated in the ALDH^+^ colorectal CSCs

**Fig. 1:**
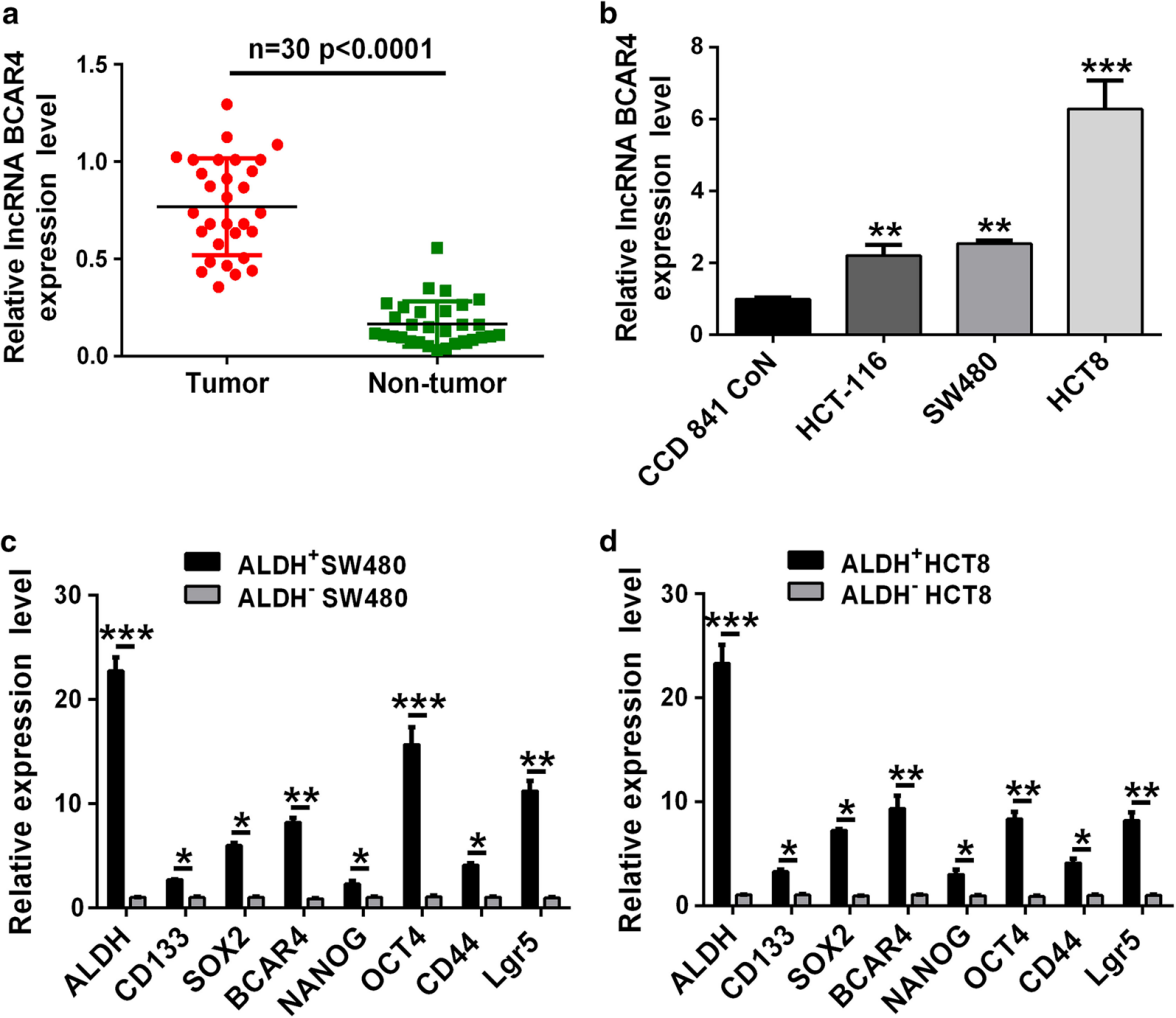
BCAR4 expression was significantly increased in CRC cells and ALDH+ cells. **a**, **b** qRT-PCR results revealed that expression of BCAR4 in CRC tissues and several CRC cell lines (HCT-116, SW480, HCT-8). **c**, **d** qRT-PCR was used to examine the expression of ALDH, CD133, SOX2, NANOG, OCT4, CD44, Lgr5 and BCAR4 in ALDH+ or ALDH− CRC cells. *P < 0.05, **P < 0.01, ***P < 0.001 vs. control

Previous studies reported that BCAR4 was highly expressed in colon cancers, however, the detailed mechanisms of BCAR4 functioned in colorectal cancer cells remains unclear. In this study, owing to the heterogeneity of colon cancer, we first examined the expression of BCAR4 in colorectal cancer tissues. As expected, qRT-PCR results showed BCAR4 expression was significantly up-regulated (Fig. 1a). In addition, immortalized colon cancer cell lines, such as HCT-116, SW480 and HCT8, were applicable to confirm the pivotal role of BCAR4 in colorectal cancer. Consistently, compared to normal colon epithelial cells (CCD 841 CoN), colorectal cancer cells exhibited high level of BCAR4 (Fig. 1b). More recent studies reported that CSCs played an important role in cancer initiation, recurrence and metastasis. Therefore, we hypothesized that whether BCAR4 could regulated CSCs in colon cancer. ALDH+ CSCs were sorted and cultured for the examination of BCAR4 expression. Consistently higher expression of CD133, SOX2, NANOG, OCT4, CD44 and Lgr5 in ALDH+ cells, which was the representative markers of CSC, validated the truth of stemness of ALDH+ colon cancer cells. More importantly, results also showed the expression of BCAR4 in ALDH+ colon cancer cells (from both SW480 and HCT8) was significantly higher than ALDH− cells (Fig. 1c, d). These results indicated ALDH+ cells occupied the capacity of stem-like cells and BCAR4 might be closely associated with CSCs stemness.

### BCAR4 promoted the stemness of colorectal CSCs

**Fig. 2:**
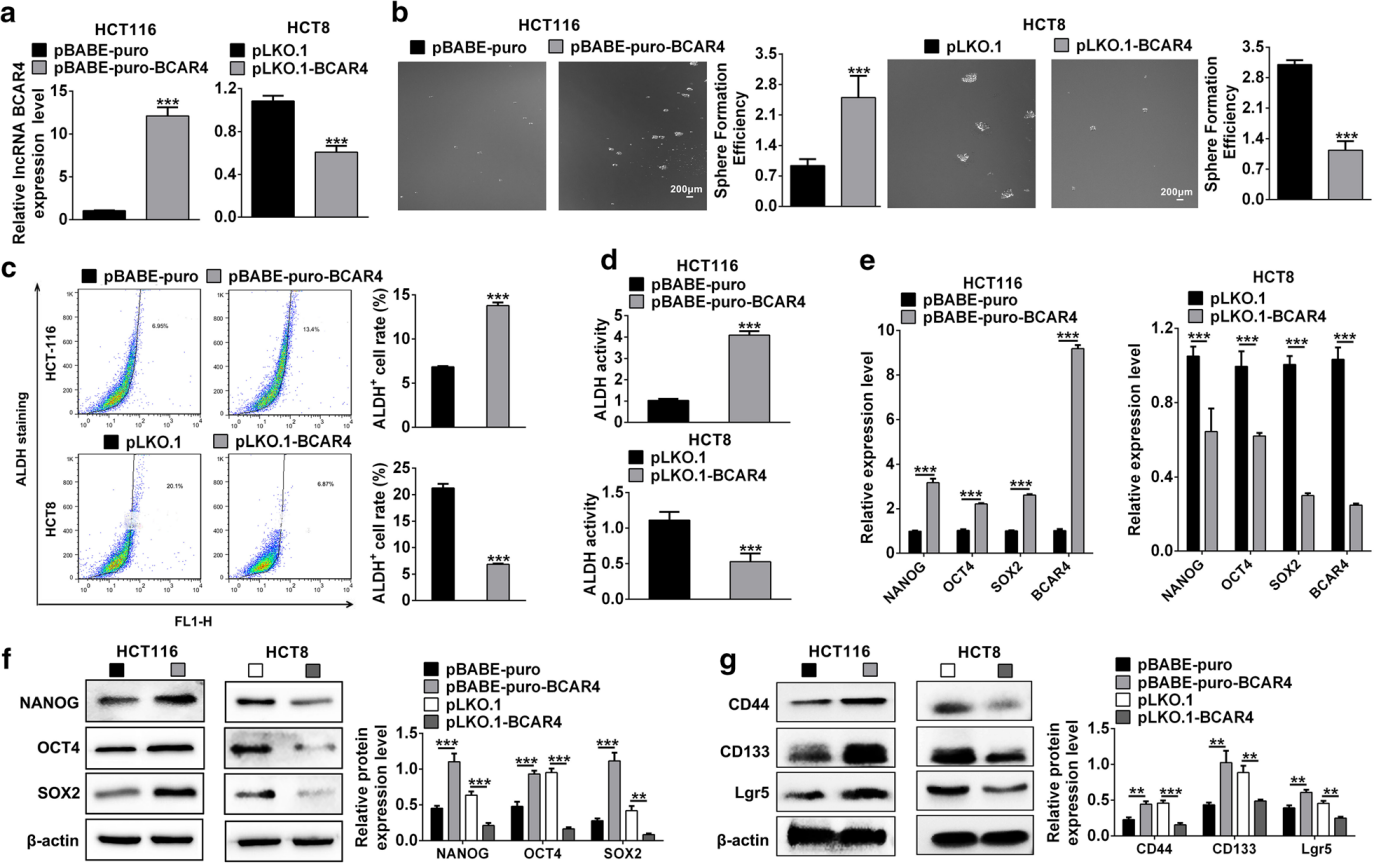
Over-expression of BCAR4 facilitated the maintenance of CSCs stemness in CRC. **a** HCT116 cells and HCT-8 cells were transfected with pBABE-puro-BCAR4 plasmid and pLKO.1-BCAR4 respectively. Transfection efficiency was examined by RT-PCR. **b** Tumorsphere formation assay revealed that BCAR4 promoted CRCs self-renewal and inhibition of BCAR4 impaired the capacity of HCT-8 cells self-renewal. **c** FACS assay showed HCT-116 cells transfected with pBABE-puro-BCAR4 plasmid increased the population of ALDH+ cells and inhibition of BCAR4 decreased the population of ALDH+ cells. **d** ALDH activity assay was used to determine the stemness alteration after BCAR4 overexpression or inhibition. **e** qRT-PCR results showed the expression of CSCs markers, such as NANOG, OCT4 and SOX2 in HCT116 cells with BCAR4 overexpression or inhibition. **f**, **g** Western blot was adopted to examine the expression of CSC markers after BCAR4 overexpression or inhibition. ***P < 0.001 vs. control

To in-depth study the function of BCAR4 in CSCs, we constructed the stable colorectal cancer cells (HCT-116-pBABE-puro-BCAR4 and HCT-8-pLKO.1-BCAR4) with BCAR4 over-expression or inhibition (Fig. 2a). Sphere formation assay revealed that colon cancer cells with higher expression of BCAR4 occupied stronger capacity of self-renewal (Fig. 2b). In addition, FACS results showed over-expression of BCAR4 increased the population of ALDH+ cells and the opposite is true (Fig. 2c). Consistently, ALDH activity assay also showed the expression of BCAR4 is positively associated with ALDH activity (Fig. 2d). Moreover, western blot and qRT-PCR results showed BCAR4^high^ cells showed higher expression of NANOG, OCT4, SOX2, CD44, CD133 and Lgr5 which are important markers of CSC in colon cancer. In contrast, inhibition of BCAR4 significantly decreased the expression of NANOG, OCT4, SOX2, CD44, CD133 and Lgr5 (Fig. 2e). These results indicated that BCAR4 positively regulated colorectal CSCs stemness.

### Inhibition of BCAR4 ameliorated CRC malignancy

**Fig. 3:**
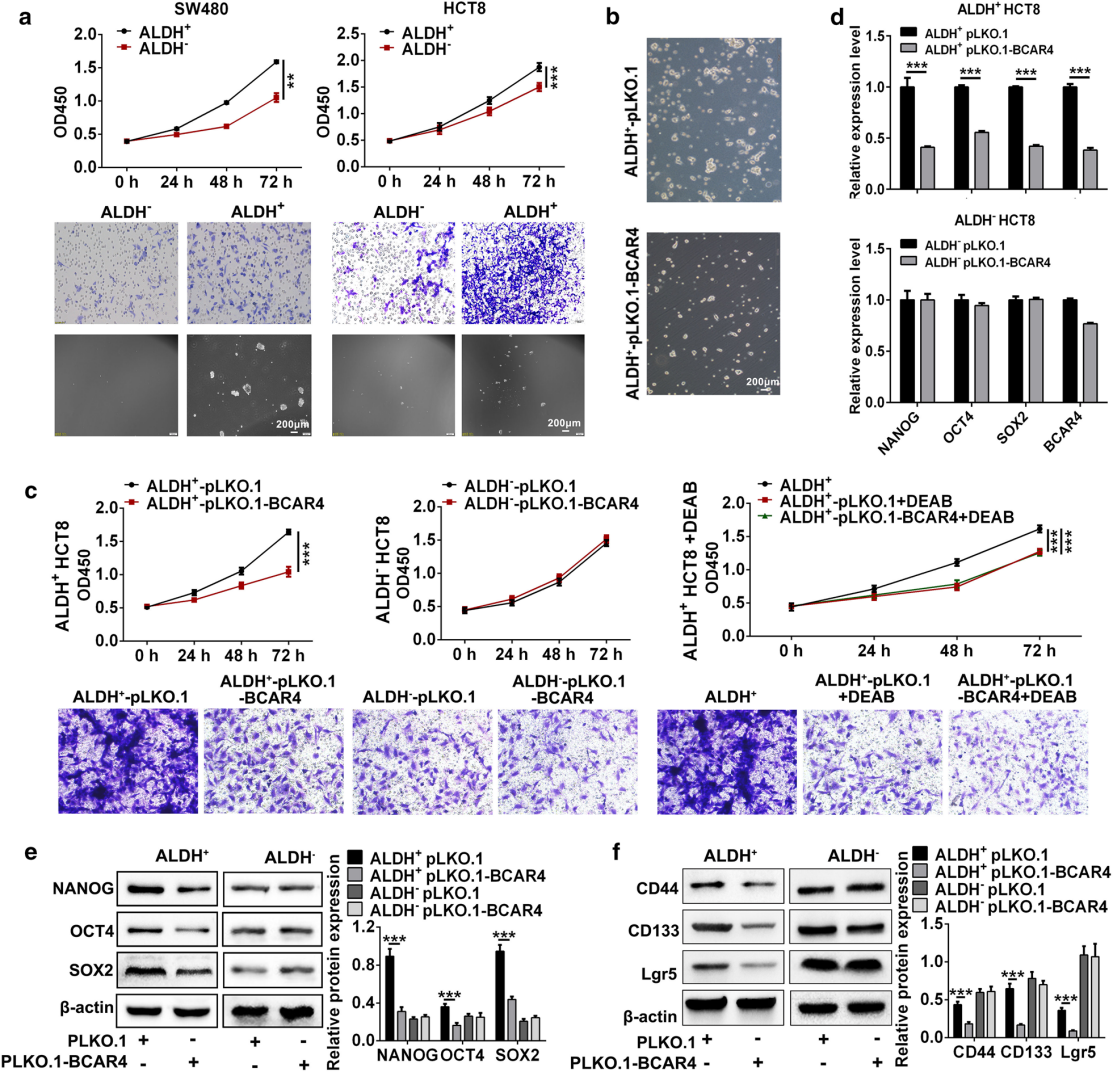
BCAR4 facilitated ALDH positive cells viability, migration and self-renewal in CRC. **a** CCK8, migration and tumorsphere formation assay revealed that ALDH+ CRC cells occupied enhanced capacity of proliferation, migration and self-renewal. **b**, **c** CCK8, migration and tumorsphere formation assay revealed the alteration of capacity of proliferation, migration and self-renewal in colon cancer cells with defected BCAR4 function. **d** q-PCR showed the expression of CSCs markers in ALDH+ or ALDH− HCT8 cells after BCAR4 inhibition. **e**, **f** Western blot showed the expression of CSC markers n ALDH+ or ALDH− HCT8 cells after BCAR4 inhibition

To further investigate the role of ALDH+ CSC in CRC. We sorted ALDH+ SW480 and HCT8 cells and found that ALDH positive CRC cells occupied higher viability compared to ALDH negative cells. In addition, enhanced ability of migration and sphere formation were observed (Fig. 3a). Considering the important role of BCAR4 in colon CSCs, we subsequently examined the potency of BCAR4 inhibition in the CRC prophylaxis and treatment. At first, we constructed stable CRC cells (HCT-8) with BCAR4 inhibition. Tumorsphere assay revealed that knock down of BCAR4 impaired CSCs stemness (Fig. 3b). Then migration assay indicated inhibition of BCAR4 impaired the migration capacity of ALDH+ cells rather than ALDH+ CRC cells. Consistently, knock down of BCAR4 showed vast effect on ALDH positive CRC cells viability but not ALDH- cells. 4-Diethylaminobenzaldehyde (DEAB), an inhibitor of ALDH, was also applicable to down-regulate ALDH activity. Similarly, DEAB significantly inhibited ALDH+ CRC cells viability and migration. However, DEAB treatment seems to have no effect on CRC cells with BCAR4 inhibition. It could be the activity of ALDH had been inhibited by PLKO.1-BCAR4 (Fig. 3c). Besides, expression of CSCs markers was also down-regulated by BCAR4 inhibition in ALDH+ cancer cell (Fig. 3d–f).

### BCAR4 regulates the malignancy of CRC through miR-665/STAT3 signaling

**Fig. 4:**
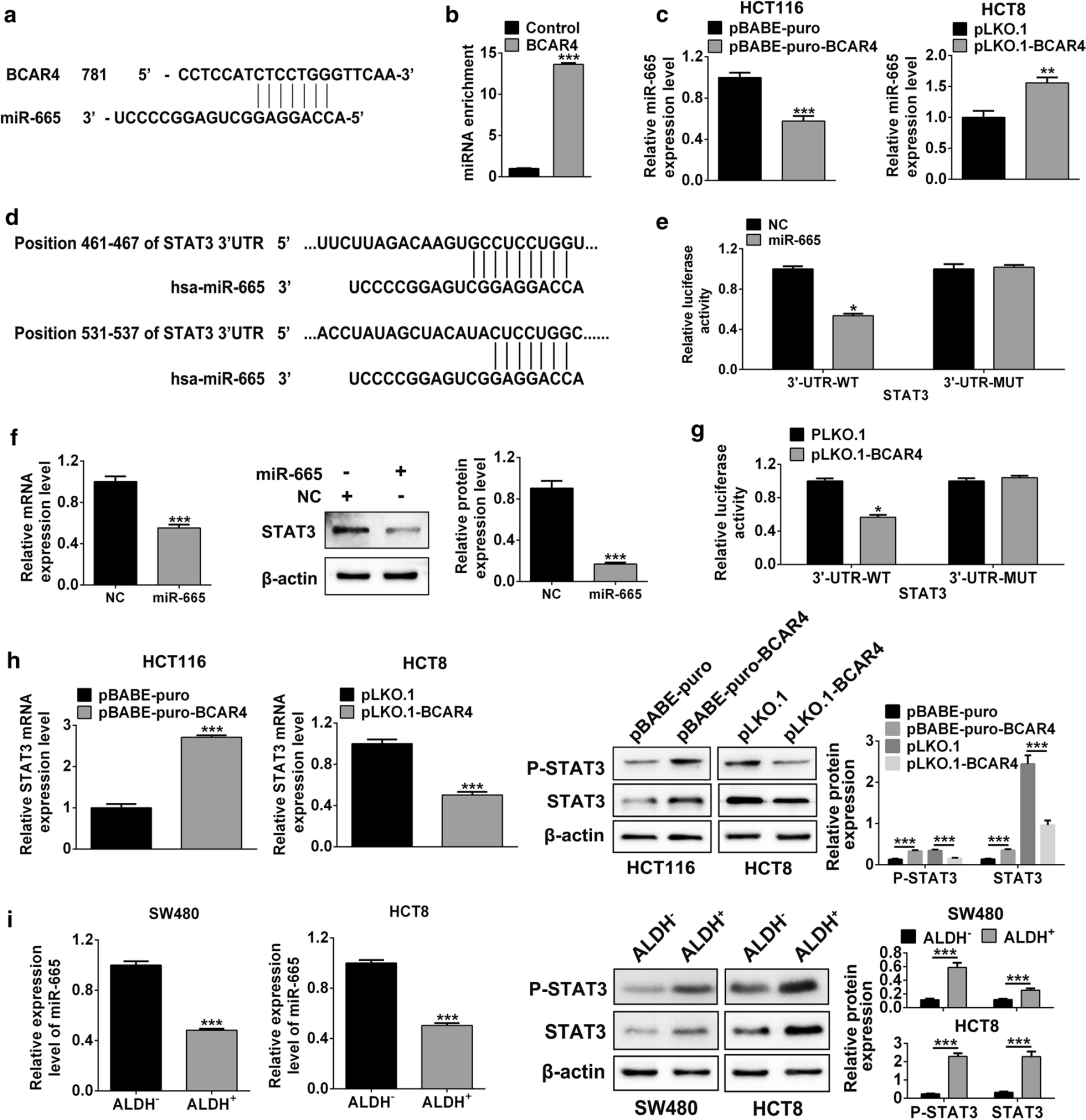
BCAR4-miR-665-STAT3 ceRNA network was functioned in CRC cells. **a** Starbase v2.0 was used to screen the potential interaction between BCAR4 and miR-665. **b** RNA pull down and qRT-PCR assay showed the enrichment of miR-665. **c** qRT-PCR was applicable to test the expression of miR665. **d** Targetscan was used to predict the interaction of miR-665 and STAT3. **e** Luciferase reporter assay was used to examine the interaction between miR-665 and STAT3. **f** qRT-PCR and western blot revealed the expression of STAT3 after miR-665 overexpression **g** luciferase reporter assay indicated the interaction between miR-665 and STAT3 after BCAR4 inhibition. **h** In CRC cells, qRT-PCR and western blot showed BCAR4 inhibition impaired the expression and phosphorylation of STAT3. **i** In ALDH+ CRC cells, qRT-PCR and western blot indicated miR-665 was down-regulated and STAT3 expression and phosphorylation was enhanced. *P < 0.05, **P < 0.01, ***P < 0.001 vs. control

**Fig. 5:**
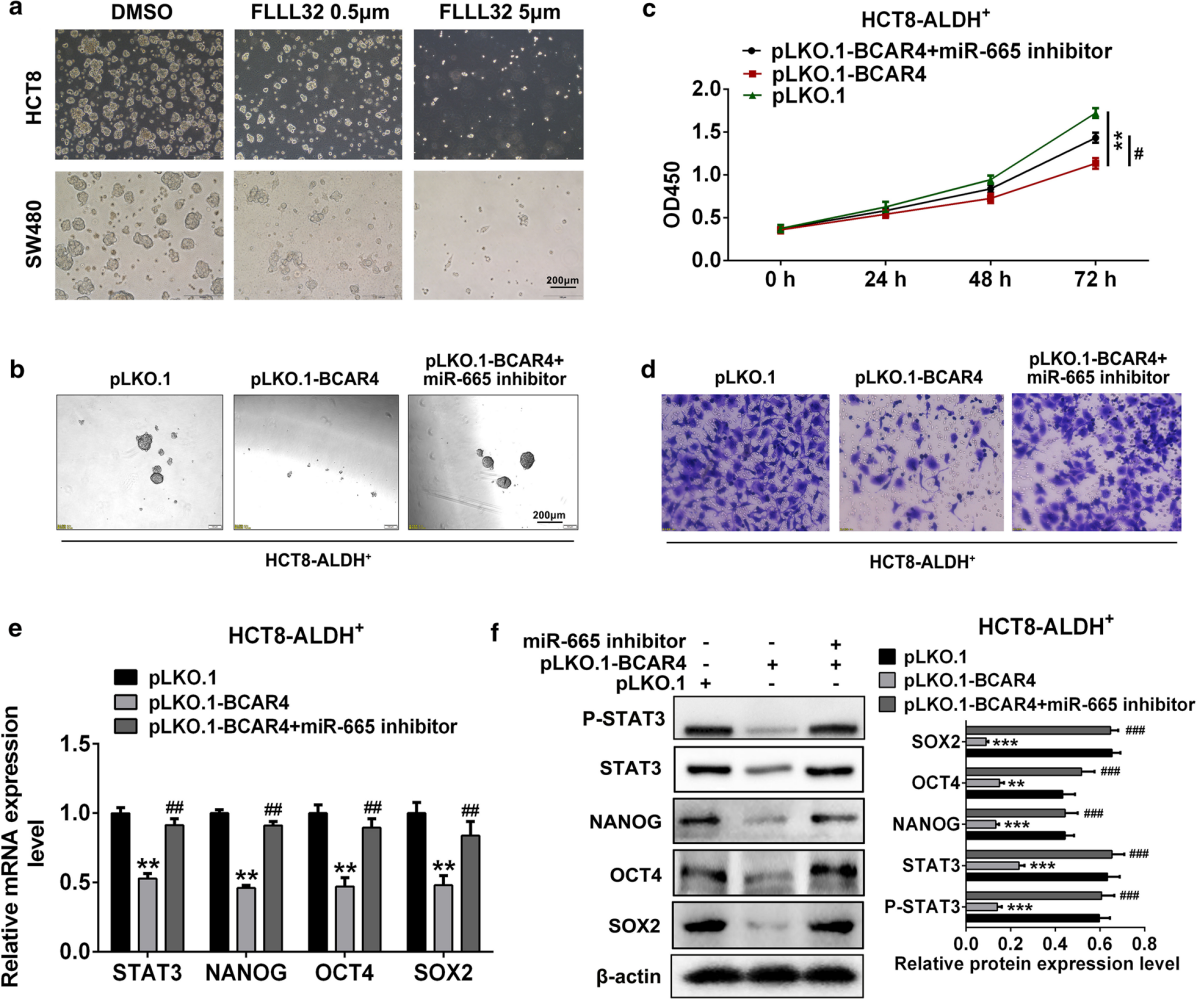
BCAR4 promoted CRC cells proliferation, migration and self-renewal through repressing miR-665. **a** Tumorsphere formation assay was used to examine the stemness of colon cancer cells after inhibition of STAT3. **b**–**d** Sphere formation, CCK8 and transwell migration assays were used to check the capacity of self-renewal, proliferation and migration after BCAR4 inhibition or miR-665 inhibition plus BCAR4 defect. **e**, **f** QRT-PCR and western blot showed the difference in expression of CSC markers and STAT3 after BCAR4 inhibition or loss of function of both BCAR4 and miR-665

To further investigate the downstream signaling regulating CRC malignancy, we screened the target genes of BCAR4 using Starbase v2.0 and found that miR-665, which is a critical regulator in cancers (23,24), was a promising target of BCAR4 (Fig. 4a). RNA pull down assay confirmed the interaction of BCAR4 and miR-665 (Fig. 4b). Furthermore, in CRC cells with BCAR4 over-expression or inhibition, miR-665 expression was negatively regulated by BCAR4 and confirmed the inhibited efficiency of BCAR4 in miR-665 expression (Fig. 4c). Next, Targetscan was used to predict the target genes of miR-665. Among all the target genes, we focused on the stat3 (Fig. 4d). As we known, STAT3 is an important transcription factor which is essential in maintenance of the stem cells stemness and is also closely associated with colon cancer. Therefore, we hypothesized that whether STAT3 is responsible for CSCs self-renewal. Subsequently, in HCT-8 cells, luciferase reporter assay and western blot assay both indicated STAT3 was a direct target gene of miR-665 (Fig. 4e, f). Additionally, in BCAR4-overexpressing cells, miR-665 was also efficiently interacted with STAT3 and high expression of BCAR4 promoted STAT3 expression and phosphorylation. On the contrary, BCAR4 inhibition also restricted the expression of STAT3 and phosphorylation of STAT3 (Fig. 4g, h). As expected, in ALDH+ colon cancer cells, miR-665 was down-regulated but STAT3 and p-STAT3 was up-regulated (Fig. 4i). FLLL32 which is an inhibitor of STAT3 signaling was supplemented in the HCT8 and SW480 cells during the tumorsphere formation assay. Results showed inhibition of STAT3 signaling repressed the capacity of CSC stemness (Fig. 5a).

To further confirm that miR-665 was negatively regulated by BCAR4, we constructed the stable ALDH+ colon cancer cells with BCAR4 inhibition. After exogenous expression of miR-665 inhibitor, the capacities of colon cancer cells sphere formation, viability and migration were reversed (Fig. 5b–d). In addition, expression of CSC markers was also increased after miR-665 inhibition and indicated BCAR4 occupied the inhibited effect on miR-665 expression (Fig. 5e, f).

### Loss-of-function (LOF) BCAR4 inhibited colorectal cancer malignancy in vivo

**Fig. 6:**
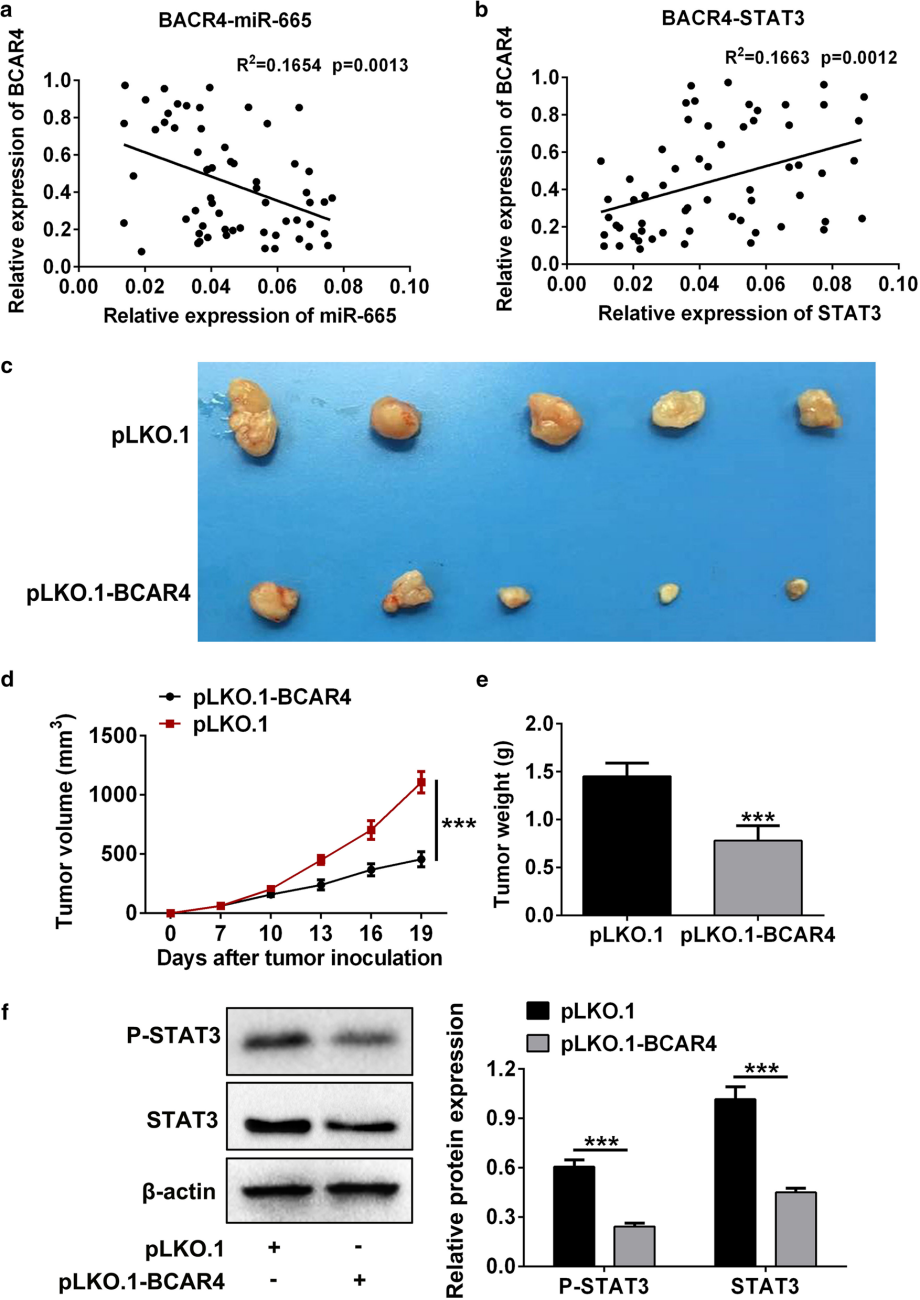
Inhibition of BCAR4 prevented tumor growth in vivo. **a**, **b** Statistical analysis was applied to evaluate the correlation between BCAR4, miR-665 and STAT3. **c**–**e** In vivo tumor formation assay was applied to examine the malignancy of CRC cells with BCAR4 inhibition. **f** Western blot was used to examine the expression and phosphorylation of STAT3 in tumor tissues with BCAR4 inhibition. ***P < 0.001 vs. control

Evidence so far suggested that BCAR4 inhibited miR-665 expression and further promoted STAT3 expression, this signaling cascade was essential to colon cancer cells survival, self-renewal and migration. Therefore, we collected 60 CRC samples to confirm this network is work in vivo. Results showed in CRC tissues, BCAR4 is negatively correlated with miR-665 and positively correlated with STAT3 (Fig. 6a, b). In addition, in vivo tumor formation assay showed BCAR4 inhibition could effectively restricted in vivo tumor growth (Fig. 6c–e). Further, knockdown of BCAR4 decreased the expression and phosphorylation of STAT3 in vivo.

Overall, these results indicated BCAR4 and STAT3 could be the novel target for CRC therapy.

## Discussion

Colorectal cancer was a devastating disease with poor prognosis and increased mortality. Diagnostic markers of early detection and later therapy process were extremely urgent to identify. Peluso et al. [18] indicated that there were three biomarkers for CRC, i.e., microsatellite instability (MSI), insulin-like growth factor binding protein 2 (IGFBP2), telomerase and pyruvate kinase M2 were responsible early CRC detection. KRAS and BRAS mutations might indicate higher risk of CRC [19]. Additionally, identification of Activated protein C (APC), P53, epidermal growth factor receptor (EGFR) and vascular endothelial growth factor (VEGF) was valuable for CRC prognosis [20]. Recent studies revealed that lncRNA BCAR4 was highly expressed in the CRC tissues. Over-expression of BCAR4 promoted CRC cells proliferation and migration through activating wnt/β-catenin signaling pathway [16]. However, the underlying mechanisms of BCAR4 dysfunction in CRC were still unclear.

In this study, we further investigate the target cells subpopulation and downstream effectors of BCAR4. Wang et al. [21] reported that BCAR4 increased cisplatin resistance in gastric cancer and predicted poor survival in several cancer types. As we known, CSCs, unlike the adult or embryonic stem cells, are thought to initiate tumor growth and cause the recurrence of cancer after chemotherapy and/or radiation therapy. Thus we hypothesized that whether BCAR4 regulated cancer progression through affecting CSC capacity and how. CSCs, depended on cancer types, occupied different markers, such as, SOX2, CD133, epithelial cell adhesion molecule (EpCAM), CD166, CD24, CD29, Lgr5, Kruppel-like factor 4 (Klf4) and ALDH [22]. We sorted ALDH positive CRC stem-like cells and examined the expression of BCAR4. Interestingly, we found that BCAR4 was significantly upregulated in the ALDH+ cells and the expression level of BCAR4 was consistent with CD133 and SOX2 which were the markers of CSC in CRC. This result indicated that BCAR4 might be closely associated with CSC stemness. Therefore, we next investigate the effect of BCAR4 on CSC tumorsphere formation and markers expression. Expectedly, after over-expression of BCAR4 in CRC cells, the capacity of sphere formation and CSCs markers expression were significantly up-regulated. In contrast, inhibition of BCAR4 impaired the self-renewal of CSCs. Yang et al. [23] indicated that BCAR4 promoted lung cancer cells viability and migration, so we subsequently examined the effect of BCAR4 on CRC cells viability and migration. Results showed that BCAR4 also promoted CSCs viability and migration in CRC. Given the important role of BCAR4 in CRC, we intended to find the target genes of BCAR4 in colon cancer. We screened several target genes of BCAR4 and found miR-665 was most attractive. MiR665 was proved to be involved in the several cancer types including prostate cancer and gastrointestinal stromal tumors [24, 25]. Moreover, recent studies revealed that lncRNA-associated competing endogenous RNA network, i.e., lncRNA–miRNA–mRNA ceRNA network, participated into the progress of CRC [26]. This concept provided further understanding in CRC pathogenesis and diagnosis. Therefore, we first examined the interaction between BCAR4 and miR-665 and found miR-665 was a target of BCAR4. Then we screened the functional genes of miR-665 and found STAT3 can function as a target of miR-665. STAT3, an important transcription factor, is closely associated with stem cells stemness [27]. In this study, we further demonstrated that STAT3 was also critical to CSCs stemness. Inhibition of STAT3 significantly inhibited the sphere formation of CSCs in colon cancer.

Evidence so far suggested that BCAR4-miR-665-STAT3 ceRNA network is responsible CRC malignancy in vitro. Therefore, we intended to investigate whether this network is work in vivo. Correlation analysis revealed that BCAR4 is negatively correlated with miR-665 and positively correlated with STAT3. In addition, inhibition of BCAR4 significantly prevented tumor growth.

## Conclusion

Overall, for the first time, we revealed that BCAR4 was essential in CRC through regulating CSC capacities. In addition, BCAR4-miR-665-STAT3 ceRNA network might also function and could be a diagnostic marker of CRC as well as provided a therapeutic target in colon cancer treatment.

## Abbreviations

BCAR4: breast cancer anti-estrogen resistance 4
CRC: colorectal cancer
ALDH: acetaldehyde dehydrogenase
STAT3: activators of transcription
CSCs: cancer stem cells
ABCB5: ATP-binding cassette sub-family B member 5
BCG: Bacillus Calmette–Guérin
LncRNAs: long non-coding RNAs
EPIC1: epigenetically-induced lncRNA1
IGF2BP1: insulin-like growth factor 2 mRNA-binding protein 1
Lgr5: Leucine-rich repeat-containing G-protein coupled receptor 5
DEAB: 4-diethylaminobenzaldehyde
MSI: microsatellite instability
APC: activated protein C
EGFR: epidermal growth factor receptor
VEGF: vascular endothelial growth factor
EpCAM: epithelial cell adhesion molecule
Klf4: Kruppel-like factor 4
